# mTORC1 Down-Regulates Cyclin-Dependent Kinase 8 (CDK8) and Cyclin C (CycC)

**DOI:** 10.1371/journal.pone.0126240

**Published:** 2015-06-04

**Authors:** Daorong Feng, Dou Yeon Youn, Xiaoping Zhao, Yanguang Gao, William J. Quinn, Alus M. Xiaoli, Yan Sun, Morris J. Birnbaum, Jeffrey E. Pessin, Fajun Yang

**Affiliations:** 1 Division of Endocrinology, Department of Medicine, Diabetes Research Center, Albert Einstein College of Medicine, New York, New York, United States of America; 2 Department of Nuclear Medicine, Renji Hospital, Shanghai Jiao Tong University School of Medicine, Shanghai, China; 3 School of Life Science and Technology, Harbin Institute of Technology, Harbin, China; 4 Division of Endocrinology, Diabetes, and Metabolism, Department of Medicine, University of Pennsylvania, Philadelphia, Pennsylvania, United States of America; 5 Department of Geriatrics, Zhongshan Hospital of Fudan University, Shanghai, China; 6 Department of Molecular Pharmacology, Albert Einstein College of Medicine, New York, New York, United States of America; 7 Department of Developmental & Molecular Biology, Albert Einstein College of Medicine, New York, New York, United States of America; INRA, FRANCE

## Abstract

In non-alcoholic fatty liver disease (NAFLD) and insulin resistance, hepatic *de novo* lipogenesis is often elevated, but the underlying mechanisms remain poorly understood. Recently, we show that CDK8 functions to suppress *de novo* lipogenesis. Here, we identify the mammalian target of rapamycin complex 1 (mTORC1) as a critical regulator of CDK8 and its activating partner CycC. Using pharmacologic and genetic approaches, we show that increased mTORC1 activation causes the reduction of the CDK8-CycC complex *in vitro* and in mouse liver *in vivo*. In addition, mTORC1 is more active in three mouse models of NAFLD, correlated with the lower abundance of the CDK8-CycC complex. Consistent with the inhibitory role of CDK8 on *de novo* lipogenesis, nuclear SREBP-1c proteins and lipogenic enzymes are accumulated in NAFLD models. Thus, our results suggest that mTORC1 activation in NAFLD and insulin resistance results in down-regulation of the CDK8-CycC complex and elevation of lipogenic protein expression.

## Introduction

Regulation of *de novo* lipogenesis in the liver is a complex process that is dependent upon the levels of nutrients and hormones, transcriptional control of lipogenic gene expression, allosteric regulation of key enzymatic activities and availability of substrates in hepatocytes [1, 2]. Sterol regulatory element-binding protein-1c (SREBP-1c) and carbohydrate responsive element-binding protein (ChREBP) are two major transcription factors that critically activate the transcription of the rate-limiting enzymes for biosynthesis of fatty acids and triglycerides [1]. *In vivo*, *de novo* lipogenesis takes place primarily in hepatocytes. SREBP-1c is the primary effector of insulin-induced *de novo* lipogenesis in hepatocytes [3], while ChREBP is mainly activated by carbohydrates [4]. Insulin and feeding acutely stimulates SREBP-1c by increasing 1) the *SREBP-1c* transcript [5, 6]; 2) the proteolytic maturation from its precursor that is initially located in endoplasmic reticulum (ER) membrane [7, 8]; and 3) the stability of nuclear SREBP-1c proteins [9, 10].

The *srebf1* gene generates two SREBP-1 isoforms (SREBP-1a and SREBP-1c) through two distinct promoters that only differ by several amino acids at the N-terminus [11, 12]. The *SREBP-1c* transcript is the predominant product of the *srebf1* gene in the liver, adrenal gland, brain and adipose tissue, while the *SREBP-1a* transcript is relatively more abundant in the spleen, macrophage and in some cancer cell lines [13–15]. A previous study has identified GSK-3β as a kinase that phosphorylates nuclear SREBP-1a/1c proteins thereby creating a recognition site for the E3 ligase SCF^Fbw7^ that ubiquitinates nuclear SREBP-1, resulting in the proteasome-mediated degradation [9]. More recently, we have shown that CDK8, a subunit of the conserved transcriptional cofactor called the Mediator complex [16–18], also phosphorylates nuclear SREBP-1c *in vitro* and *in vivo* on the conserved T402 residue to promote ubiquitination and degradation thereby suppressing expression of SREBP-1-target genes [10]. As a result, CDK8 and CycC deficiency in fruit flies and mice resulted in increased lipogenic gene expression, increased *de novo* lipogenesis and lipid accumulation [10]. Physiologically, the CDK8 and CycC protein levels in mouse liver were lower in the fed state as compared to the fasted state, consistent with the accumulation of nuclear SREBP-1c to induce lipogenic gene expression during feeding and the decrease of nuclear SREBP-1c during fasting [10]. Moreover, we showed that insulin could down-regulate the CDK8 and CycC proteins in isolated primary rat hepatocytes [10]. Thus, the nutrient/hormone regulation of the CDK8-CycC complex functionally regulates the nuclear SREBP-1c protein levels as a part of the complex mechanisms controlling hepatic lipogenic gene expression.

In the postprandial state, the combination of increased plasma nutrients and insulin levels results in the stimulation of liver *de novo* lipogenesis for energy storage while suppressing gluconeogenesis to prevent the development of hyperglycemia [19]. However, in insulin resistant states, the ability of insulin to suppress gluconeogenesis is blunted, whereas insulin remains able to activate *de novo* lipogenesis, resulting in the persistent activation of both *de novo* lipogenesis and gluconeogenesis [19]. The molecular basis of this apparent selective insulin resistance has remained enigmatic and is a major metabolic concern for understanding and treatment of non-alcoholic fatty liver disease (NAFLD) and type 2 diabetes [19]. In this study, using genetic and aging mouse models of insulin resistance and NAFLD, we show that mTORC1-dependent down-regulation of the CDK8-CycC complex plays a role in the persistent activation of *de novo* lipogenesis.

## Materials and Methods

### Antibodies

The anti-CDK8 (ab2955), anti-Cyclin C (ab2950)) and anti-Ulk1 (ab128859) antibodies were purchased from Abcam. The anti-FAS (#8023), anti-S6K1 (#2708), anti-phosph-T389/S6K1 (#9234), anti-phosph-S235/236/S6 (#4858), anti-Raptor (#2280), anti-SCD1 (#2283), anti-phosph-S757/Ulk1 (#6888) antibodies were purchased from Cell Signaling, and anti-TBP antibody (#51841) antibody from Fisher Scientific, anti-β-tubulin (PA1-21153) antibody from Life Technologies, anti-CDK8 (SC-1521) and anti-SREBP-1 (SC-13551) antibodies from Santa Cruz and anti-β-actin (AA2-033) antibody from Sigma. The specific anti-phospho-T402-SREBP-1 antibody was generated by Genescript.

### Tissue Culture

HEK293T and FAO cells were purchased from ATCC and cultured in Dulbecco’s modified Eagle’s medium (Life Technologies) supplemented with 10% heat-inactivated fetal bovine serum (Hyclone), 2 mM L-Glutamine (Life Technologies), 100 units/ml penicillin-streptomycin (Life Technologies) at 37°C under humidified air containing 5% CO_2_.

### Protein Extraction and Immunoblotting

For whole cell extracts, cells or homogenized mouse tissues were lysed in a buffer containing 50 mM Hepes (pH = 8.0), 1mM EDTA, 150 mM NaCl, 1% Triton X-100, 2mM Na_3_VO_4_, 20mM Na_4_P_2_O_7_, 100mM NaF, 10% Glycerol, 1 mM dithiothreitol, 2.5mM PMSF, 1mM benzamidine, 1 mg/l aprotinin, and 0.1 mM ALLN. Supernatants were collected after centrifugation at 1.4×10^4^ rpm for 20 min at 4°C. Protein concentrations were measured with a BCA kit (Pierce). A given amount of whole cell extracts was mixed with 5×SDS loading buffer (0.25M Tris-HCl pH = 6.8, 10% SDS, 50% glycerol, 0.05% bromphenol blue, 500 mM dithiothreitol). After boiling for 3 min, the proteins were resolved by NuPAGE 4–12% Bis-Tris gel (Life Technologies) and transferred to nitrocellulose or PVDF membrane by iBlot Gel Transfer Kit (Life Technologies). After blocking in 5% non-fat milk in 1×TBST, the membrane was incubated with specific primary antibodies with appropriate dilution overnight at 4°C and washed three times with 1×TBST (10 min each). Then, the membrane was incubated with the HRP-conjugated secondary antibodies (1:10,000 dilution) for 1 hour at room temperature. The HRP signals were visualized by the SuperSignal West Pico kit (Pierce) after three times with 1×TBST (10 min each) according to the manufacturer’s instructions. The quantification of immunoblots was performed using the Image J software.

### RNA Preparation and Quantitative RT-PCR Analysis

Total RNA was isolated from cells and mouse livers using the Trizol Reagent (Life Technologies) according to the manufacture’s protocol. RNA concentration was measured by NanoDrop spectrophotometer (ThermoScientific). After removing genomic DNA with RQ1 RNase-free DNase I (Qiagen), the first-strand cDNA was synthesized using Omniscript RT Kit with random primers and analyzed using the FastStart Universal SYBR Green Master Mix (Roche). Each real-time PCR reaction mixture contained 10μl SYBR Green Master Mix, 1μl primers (250 nM each) and 9μl 10× diluted cDNA. Real-time PCR was performed using the StepOnePlus Real-Time PCR System (Applied Biosystems). The cycling parameters consisted of 95°C incubation for 10 min for enzyme activation and DNA denaturation, followed by 40 PCR amplification cycles consisting of 95°C for 15 sec and 60°C for 1min. The thermocycling program was followed by a melting program of 95°C for 15 sec (denaturation), 60°C for 1min (annealing), and then 60–95°C at a transition rate of 0.3°C/sec with continual monitoring of fluorescence. Data analysis is performed by software provided by StepOnePlus Real-Time PCR System.

### Mice Care and Treatment

All mouse experiments conformed to the protocols approved by the Animal Care and Use Committees of Albert Einstein College of Medicine and University of Pennsylvania School of Medicine in accordance with the National Institutes of Health (NIH) guidelines. All mice were euthanized by CO_2_ asphyxiation. Male *db/db and ob/ob* mice were maintained in the C57BL/6J background for greater than 10 generations. Wild-type C57BL/6J mice at the age of 4 months were purchased from The Jackson Laboratory, and for the aging studies male C57Bl/6 aged mice were purchased from NIH. Upon arrival, mice were maintained under a 12-hour dark cycle with free access to water and standard mouse diet (~5% calorie from fat) for one week before experiments (Lab Diet #5053). Liver-specific Raptor-knockout mice were generated by tail-vein injection of male *Raptor* ^flox/flox^ mice with AAV-Cre (1×10^11^ genomic copies of viral particles) whose expression is under the control of the hepatocyte-specific thyroxine-binding globulin (TBG) promoter. As controls, the *Raptor* ^flox/flox^ mice were injected with and AAV-GFP virus. For rapamycin treatment, rapamycin (LC Laboratories) was dissolved in ethanol at a concentration of 20 mg/ml, filter-sterilized and re-suspended in vehicle (saline containing 0.25% PEG and 0.25% Tween-80) at a concentration of 1 mg/ml. Mice at the age of 12 months were intra-peritoneally injected with rapamycin (2 mg/kg body weight) or vehicle control once a day for 3 days.

### Human Liver Samples

All experiments using human liver samples conformed to the protocols approved by the Protection of Human Subjects Committee at Zhongshan Hospital (Shanghai, China) and the Committee on Clinical Investigation at Albert Einstein College of Medicine. Formalin fixed paraffin-embedded (FFPE) liver tissues were selected from a collection of liver needle biopsy specimens from Zhongshan Hospital. These liver biopsies were collected from patients for histological analyses of liver tumors. All patients provided informed, written consent for the liver tissue to be used for tumor diagnosis and research purposes. All patient information was anonymized, and only tumor-free specimens were used in this study. The total proteins were extracted from four 5-mm^2^ liver slides from one patient. FFPE slides were incubated at 60°C for 1 hour, and incubated with histological grade Xylene for 2×10min for de-paraffinization. The slides were rehydrated in graded ethanol (100%, 90%, 80%, 70%, 50%), immersed in distilled water, and air-dried. With immersing in 1×PBS, tissues were separated from glass slide and further washed with ice-cold 1×PBS for three times. After centrifugation, tissue pellets were re-suspended in the lysis buffer (50 mM Tris-HCl pH = 8.0, 0.1 mM EDTA, 420 mM NaCl, 0.5% Nonidet P-40, 2% SDS, 10% glycerol, 1 mM dithiothreitol, 2.5 mM PMSF, 1 mM benzamidine, 1 mg/l aprotinin and 0.1 mM ALLN). The tissue suspension was passed through 25-gauge needle ten times and then incubated at 100°C for 20 min, followed by incubation at 60°C for 2 hours. After incubation, the lysates were centrifuged at 1.4×10^4^ rpm for 20 min at 4°C. The supernatants were collected and stored at -80°C until immunoblotting analysis.

### Statistical Analysis

Data were presented as Mean ± S.D., and compared between two groups using Student's *t*-test. Difference was considered statistically significant, if *p*<0.05.

## Results

### The CDK8-CycC complex in the liver is down regulated in NAFLD

**Fig 1:**
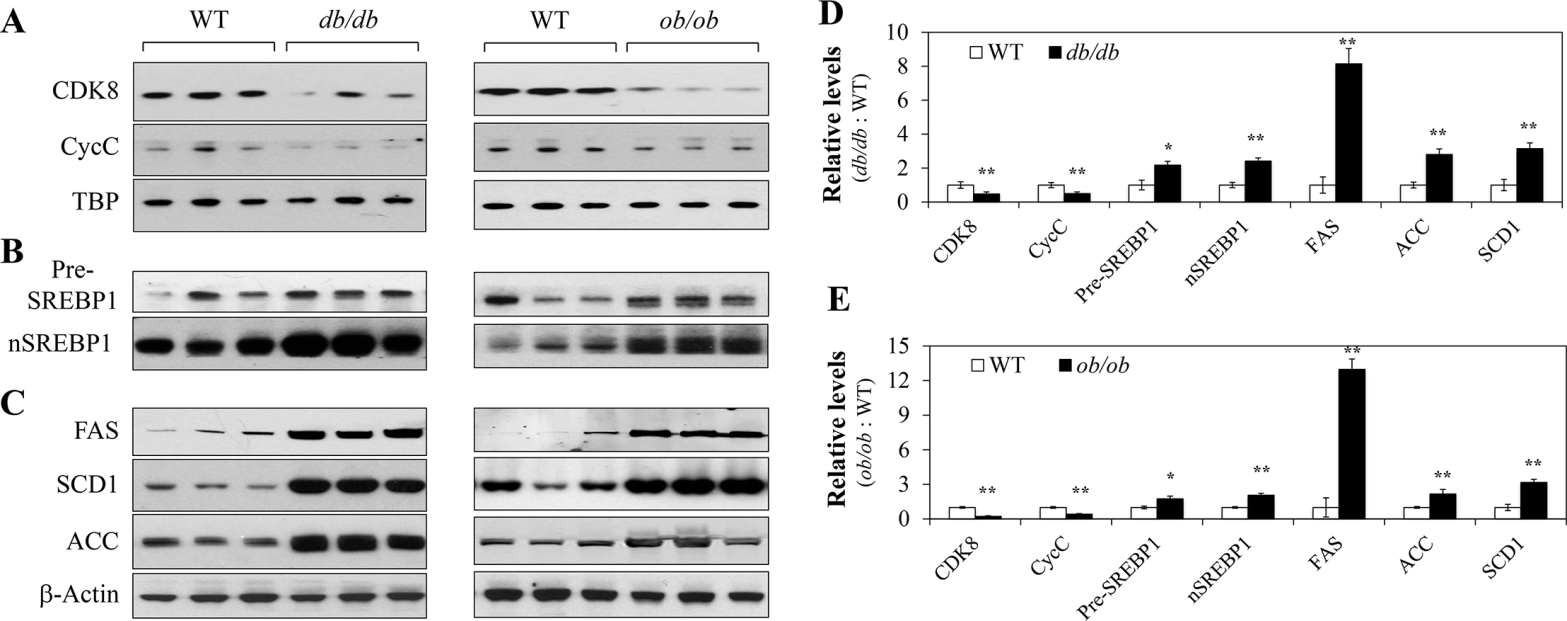
Hepatic CDK8 and CycC proteins are down regulated in genetically obese and insulin resistant mice. Control wild-type and *db/db* (left panels) or *ob/ob* (right panels) male mice on the C57Bl/6J background were maintained on the normal chow diet. At 4 months of age, these mice were fasted for 16 hours before sacrifice. **A**, **B**, **C**) Liver extracts were prepared and analyzed for the indicated proteins by immunoblotting. TBP or β-actin served as the invariant control. The SREBP-1 precursor (pre-SREBP1) and the nuclear SREBP-1 (nSREBP-1) from liver extracts of the same mice were also analyzed. Each lane represents an independent mouse. The relative protein levels in the livers of *db/db* (**D**) or *ob/ob* (**E**) mice were further analyzed by densitometry. *p<0.05 and **<0.01 vs. wild-type (n = 6 independent mice).

To examine the potential role of the CDK8-CycC complex in obesity, insulin resistance and NAFLD, we first examined the protein expression profiles in both leptin receptor-deficient (*db/db*) and leptin-deficient (*ob/ob*) mice, two well-studied models of obesity with insulin resistance and fatty liver. As shown in Fig 1A, both mouse models of obesity displayed significantly lower levels of CDK8 and CycC proteins. In parallel, the protein levels of transcriptionally active nuclear form of SREBP-1 (nSREBP-1) in the same liver samples were also significantly higher than those in wild-type mice (Fig 1B). However, the levels of the endoplasmic reticulum (ER)-bound SREBP-1 precursor (pSREBP-1) were not consistently different (Fig 1B). Although the commercial anti-SREBP-1 antibody is unable to distinguish SREBP-1c from SREBP-1a, it is likely that these immunoblots mainly represent the SREBP-1c protein, as this isoform is the dominant transcript in the liver [13]. Consistent with the significant accumulation of nSREBP-1c, both mouse models of obesity displayed significantly higher protein levels of the classical SREBP-1c-target lipogenic genes, including fatty acid synthase (FAS), stearoyl CoA desaturase 1 (SCD1) and acetyl CoA carboxylase 1 (ACC), in the liver as compared to wild-type controls (Fig 1C). In addition, the mRNA levels of these lipogenic genes were also higher in these genetically obese mice (data not shown). Image analyses of the semi-quantification of the changes in protein levels between wild-type control and *db/db* or *ob/ob* mice livers are shown in Fig 1D and 1E.

**Fig 2:**
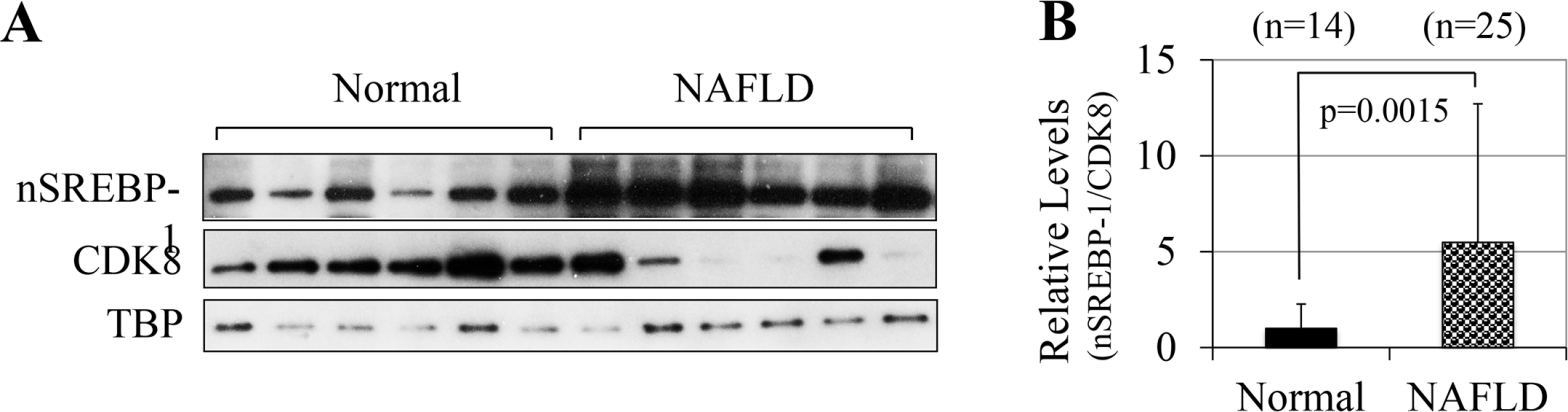
Hepatic CDK8 protein levels are inversely correlated with nuclear SREBP-1 levels in human NAFLD. **A**) Representative immunoblots of indicated proteins in total protein extracts of normal human liver biopsies (<5% fat by ultrasound analysis) or patients with diagnosed with non-alcoholic fatty liver disease, NAFLD (>30% fat). TBP served as the invariant control. **B**) The ratio of nuclear SREBP-1 (nSREBP1) to CDK8 of densitometry analyses after normalized by TBP from 14 normal and 25 NAFLD liver biopsies.

Similarly, we also observed significantly higher levels of nSREBP-1c proteins and generally lower levels of CDK8 protein in human NAFLD biopsies samples as compared to normal livers (Fig 2A). By semi-quantitatively analyzing the liver biopsies samples, we found that the ratio of nSREBP-1c to CDK8 was more than five-fold higher in NAFLD (Fig 2B), revealing a significant inverse correlation between nSREBP-1c and CDK8 protein levels in human livers. Since CDK8 negatively regulates nSREBP-1c protein degradation [10], our results of CDK8 down-regulation in NAFLD suggest that the protein stability of nSREBP-1c is increased in NAFLD, although other mechanisms of SREBP-1c regulation, such as transcription and maturation, may also contribute to the accumulation of nSREBP-1c.

### The CDK8-CycC complex levels are inversely correlated with the activity of mTORC1

**Fig 3:**
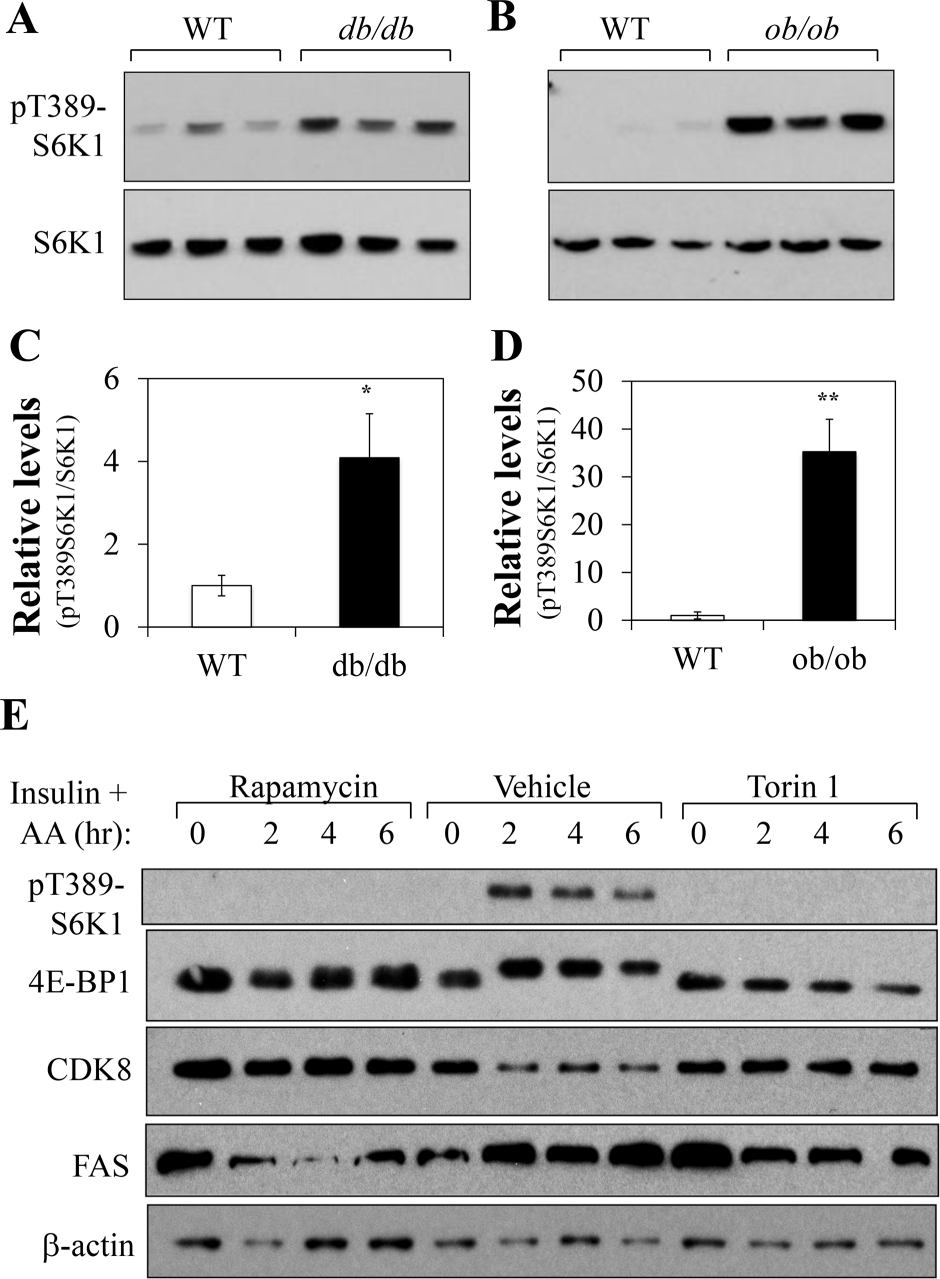
mTORC1 is activated in obese mouse livers, and is involved in nutrient-repletion-induced down-regulation of CDK8 in tissue culture. Control wild-type and *db/db* (**A**) or *ob/ob* (**B**) male mice on the C57Bl/6J background were maintained on the normal chow diet. At 4 months of age, the mice were fasted for 16 hours and then re-fed for 4 hours before sacrifice. Liver extracts were prepared and analyzed for the levels of S6K1 phosphorylation on T389 by immunoblotting. Total amount of S6K1 served as the loading control. The relative protein levels in the livers of *db/db* (**C**) or *ob/ob* (**D**) mice were further analyzed by densitometry. *p<0.05 and **<0.01 vs. wild-type (n = 3 independent mice). **E**) Effects of nutrient-depletion and nutrient-repletion on the levels of indicated proteins in the presence of mTOR inhibitor rapamycin or Torin 1. FAO cells were cultured in the low-serum DMEM overnight, and then in amino acid-free RPMI for 2 hour. The cells were pre-treated without or with 100nM rapamycin or 250nM Torin 1 for 30 min followed by the addition of regular DMEM containing 100nM insulin for the indicated period of time. Cell extracts were prepared and analyzed for the indicated proteins by immunoblotting. These are representative immunoblots independently performed for four times.

To understand the underlying mechanisms responsible for the down-regulation of CDK8 and CycC proteins, we examined the mRNA levels of *CDK8* and *CycC*, but found no significant difference (data not shown), suggesting a post-transcriptional mechanism(s) of regulating the CDK8-CycC complex under these pathophysiological conditions. Our recent study has demonstrated that feeding or insulin, which is known to activate mTORC1, down-regulates the CDK8 and CycC proteins [10]. In addition, previous studies have observed a link between insulin resistance, mTORC1 activation and increased lipogenic gene expression through activating SREBP-1c [20, 21]. As expected, both the *db/db* and *ob/ob* mice displayed increased mTORC1 activation as detected by increased phosphorylation of the mTORC1 specific site (T389) of S6 kinase 1, S6K1 (Fig 3A–3D). Therefore, we asked whether mTORC1 activation could be required for the down-regulation of the CDK8-CycC complex. For that purpose, we initially manipulated mTORC1 activation in rat hepatoma FAO cells by nutrient-deprivation and nutrient-repletion in tissue culture. For nutrient-deprivation, cells were cultured overnight in serum-free/low-glucose medium followed by additional 2 hrs of incubation in amino acid-free medium. For nutrient-repletion, cells were first fasted and then cultured in regular culture medium for various periods of time. As shown in Fig 3E, in control cells, nutrient-repletion markedly activated mTORC1 as detected by the increased T389 phosphorylation of S6K1 and the gel-shift of 4E-BP1, indicative of phosphorylation (Fig 3E, lanes 6–8 vs. lane 5). In parallel, the CDK8 protein was down regulated during this time period while the FAS protein was up regulated. As expected, pre-treatment of cells with rapamycin (Fig 3E, lanes 1–4) or Torin 1 (Fig 3E, lanes 9–12), the selective inhibitors of mTORC1 and mTOR itself, effectively suppressed mTORC1 activation. More importantly, rapamycin or Torin 1 treatment also prevented the nutrient-repletion induced down-regulation of the CDK8 proteins, supporting a role of mTORC1 in down-regulating CDK8. Interestingly, the pharmacological inhibition of mTORC1 resulted in a down-regulation of FAS protein levels suggesting that a basal activity of mTORC1 is necessary to maintain the levels of FAS proteins. In any case, the apparent mTORC1-dependent decrease of CDK8 occurred at the protein level, as there is no significant change in the *CDK8* mRNA levels (data not shown).

### mTORC1 controls the abundance of the CDK8-CycC complex in tissue culture

**Fig 4:**
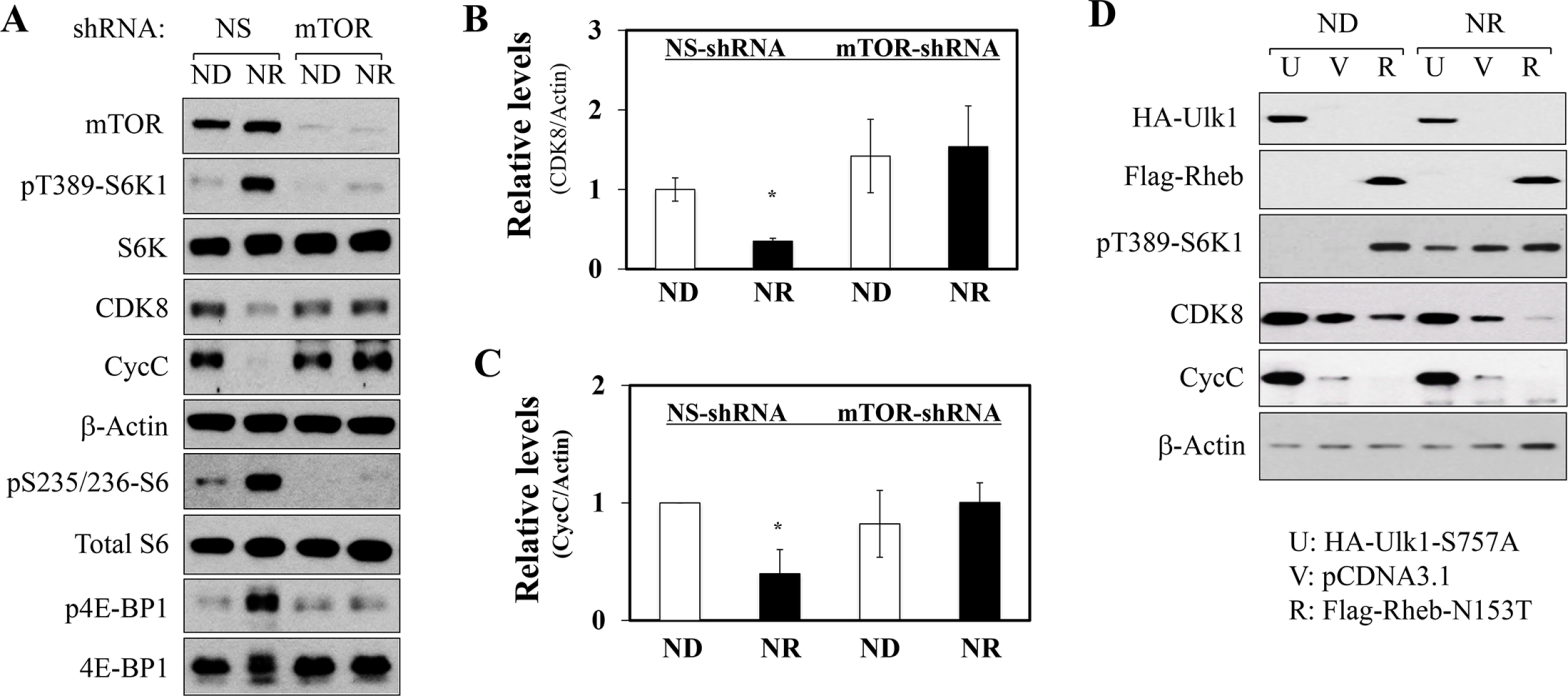
mTORC1 activity negatively regulates the CDK8 and CycC protein levels. **A**) Effects of mTOR knockdown on the levels of indicated proteins during nutrient-depletion or nutrient-repletion. HEK293T cells were infected with non-specific (NS) or mTOR-specific shRNA lentiviruses. The cells were nutrient-depleted by placing them in the low serum DMEM medium overnight, and then in amino acid-free RPMI for 2 hour followed by the addition of regular DMEM containing 100 nM insulin for 30 min. The relative protein levels of CDK8 (**B**) and CycC (**C**) were further analyzed by densitometry. *p<0.05 vs. control (n = 3). **D**) Effects of overexpressing a mutant of constitutively active Rheb or Ulk1 on the levels of indicated proteins during nutrient-depletion (ND) or nutrient-repletion (NR) condition. HEK293T cells were transfected with a constitutively active ULK1 mutant cDNA (U, 1 μg), the empty vector (V, 1 μg) or a constitutively active Rheb mutant cDNA (R, 1 μg). Thirty-six hours after transfection, the cells were nutrient-depleted and nutrient-replete as described above. These are representative immunoblots independently performed for five times.

To further define whether the mTORC1 complex regulates the CDK8 and CycC proteins, we used specific shRNAs to knockdown the mTOR catalytic subunit. As shown in Fig 4A, the first shRNA was highly effective in depleting mTOR proteins in HEK293T cells as compared to a non-specific (NS) shRNA. As expected, following stimulation with amino acids/serum, there was a dramatic increase in phosphorylation of mTORC1 downstream targets (pT389-S6K1, pT37/46-4EBP1 and pS235/236-S6) in NS-shRNA-treated cells, and the CDK8 and CycC protein levels were decreased (Fig 4A–4C). However, in mTOR-knockdown cells, there was a substantial reduction in phosphorylation of the known mTORC1 targets (Fig 4A). More importantly, mTOR knockdown prevented the nutrient-repletion induced decrease of CDK8 and CycC protein levels (Fig 4A–4C). To eliminate the potential off-target effects of shRNA, a second independent mTOR-shRNA was used and we essentially obtained identical results (data not shown), suggesting a specific effect of mTOR on down-regulating the CDK8-CycC complex.

The regulation of mTORC1 activity is controlled through multiple overlapping inputs with the small GTPase Rheb required for mTORC1 activation by stimuli, including amino acids [22, 23]. In addition, as a downstream target of mTORC1, the Ulk1 kinase induces a negative-feedback inhibition of mTORC1 by multiple mechanisms [24]. Thus, we examined whether Rheb and Ulk1 are involved in regulating CDK8 and CycC protein levels through modulation of mTORC1. To this end, a mutant of constitutively active Rheb (N153T) [25] or Ulk1 (S757A) [26] was overexpressed in HEK293T cells by transient transfection. As expected, overexpression of the constitutively active Rheb mutant activated mTORC1, while overexpression of the constitutively active Ulk1 mutant inhibited mTORC1, as detected by T389 phosphorylation of S6K1 (Fig 4D). Consistent with our data from pharmacological inhibitors or shRNA-mediated knockdown of mTOR, overexpression of Rheb or Ulk1 mutant also demonstrated a clear inverse relationship between the protein levels of the CDK8-CycC complex and the mTORC1 activation state. That is, mTORC1 activation reduced, whereas mTORC1 inhibition increased the CDK8 and CycC protein levels (Fig 4D). This inverse relationship occurred in both the nutrient-deprived and nutrient-replete states (Fig 4D).

### mTORC1 activation is required for feeding-induced down-regulation of the CDK8-CycC complex *in vivo*

**Fig 5:**
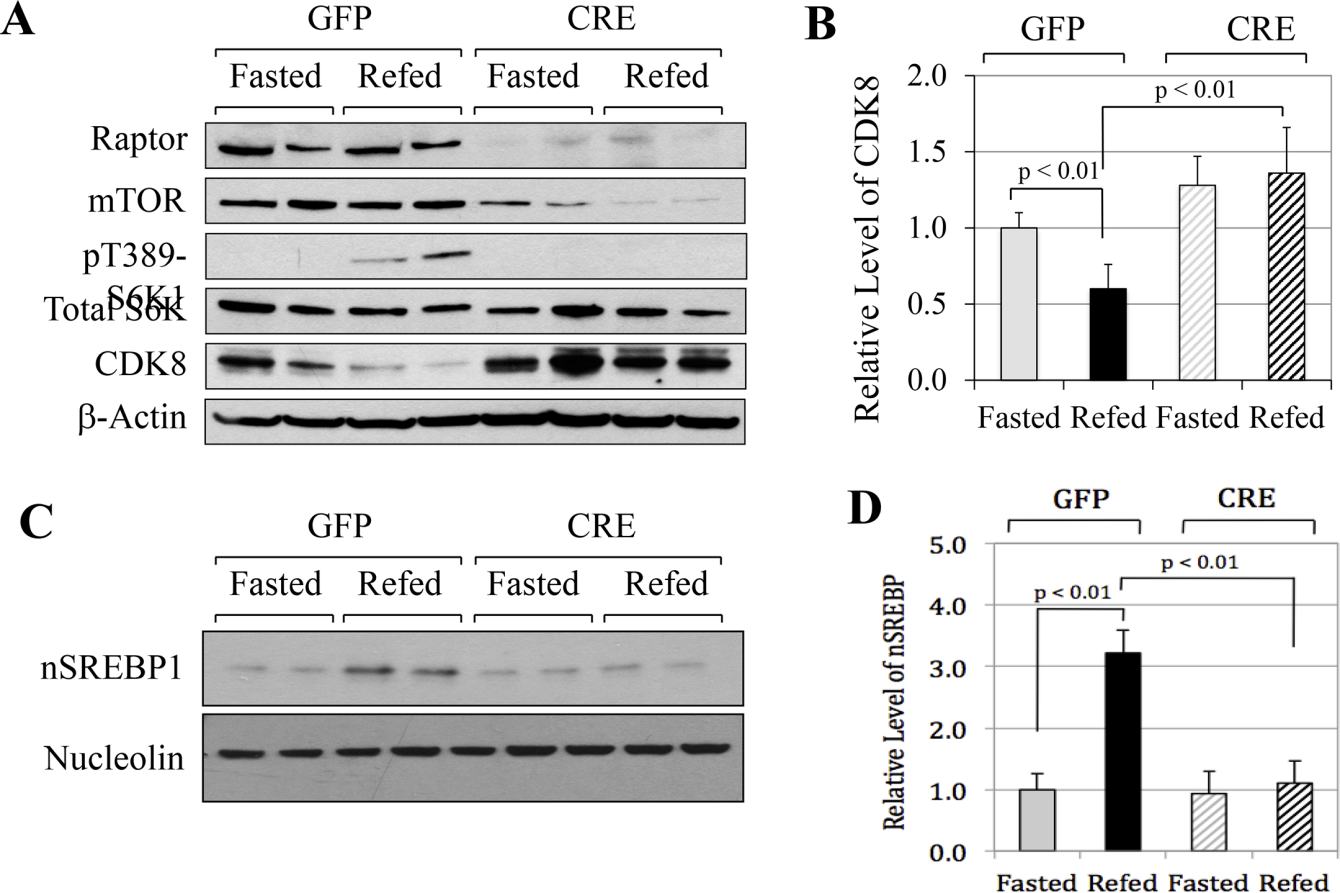
mTORC1 activation is required for feeding-induced down-regulation of CDK8. **A**) Representative immunoblots showing the levels of indicated proteins in duplicate from livers of liver-specific Raptor knockout mice or controls. Knockout was achieved by tail-vein injection of AAV-TBG-Cre (or GFP as the control) in *Raptor* ^flox/flox^ mice. One week after the AAV injections, the mice were fasted for 12 hours, and then either sacrificed or re-fed for 5 hours. **B**) The ratios of CDK8 to β-actin of indicated liver samples as analyzed by densitometry (n = 7 mice for each group). **C**) Representative immunoblots showing the levels of the indicated proteins in duplicate from livers of liver-specific Raptor knockout mice or controls as indicated. **D**) The ratios of nSREBP1 to the invariant nucleolin of the indicated liver samples as analyzed by densitometry (n = 4 mice for each group).

As a critical component of the mTORC1 complex, the regulatory-associated protein of mTOR (Raptor) aids in the substrate recognition [27]. To determine whether specific alteration of the mTORC1 activity by the genetic approaches also affects the CDK8-CycC complex *in vivo*, we examined mice with liver-specific knockout of Raptor. Depletion of Raptor in the livers of *Raptor* ^flox/flox^ mice was achieved through tail-vein injection of adeno-associated viruses (AAV) expressing either GFP (control) or Cre recombinase under the control of the hepatocyte-specific TBG promoter [28]. As shown in Fig 5A, overexpressing Cre efficiently reduced the protein levels of hepatic Raptor as compared to the GFP controls, indicating the success of Raptor knockout. Interestingly, Raptor-knockout also reduced the protein levels of mTOR. The dependence of mTOR protein levels on the Raptor protein and conversely the levels of Raptor protein dependent on mTOR levels were also observed in HEK293 cells with siRNA-mediated knockdown of mTOR or Raptor [27].

As a result, Raptor-knockout mice failed to activate mTORC1 in the re-fed state, as detected by T389 phosphorylation of S6K1, while the control mice displayed a strong activation of mTORC1 (Fig 5A). Confirming our recent report [10], re-feeding caused a significant decrease of hepatic CDK8 protein levels in the control GFP-overexpressing mice (Fig 5A and 5B). However, Raptor knockout mice displayed higher protein levels of hepatic CDK8 in both fasted and re-fed states, and re-feeding failed to down-regulate CDK8 (Fig 5A and 5B). Consistent with the role of mTORC1 in activating SREBP-1c, Raptor knockout abolished feeding-induced accumulation of nuclear SREBP-1c (Fig 5C and 5D). Thus, our data demonstrate that mTORC1 activation is required for the down-regulation of the CDK8-CycC complex during feeding *in vivo*.

### mTORC1 regulates the CDK8-CycC complex during aging

**Fig 6:**
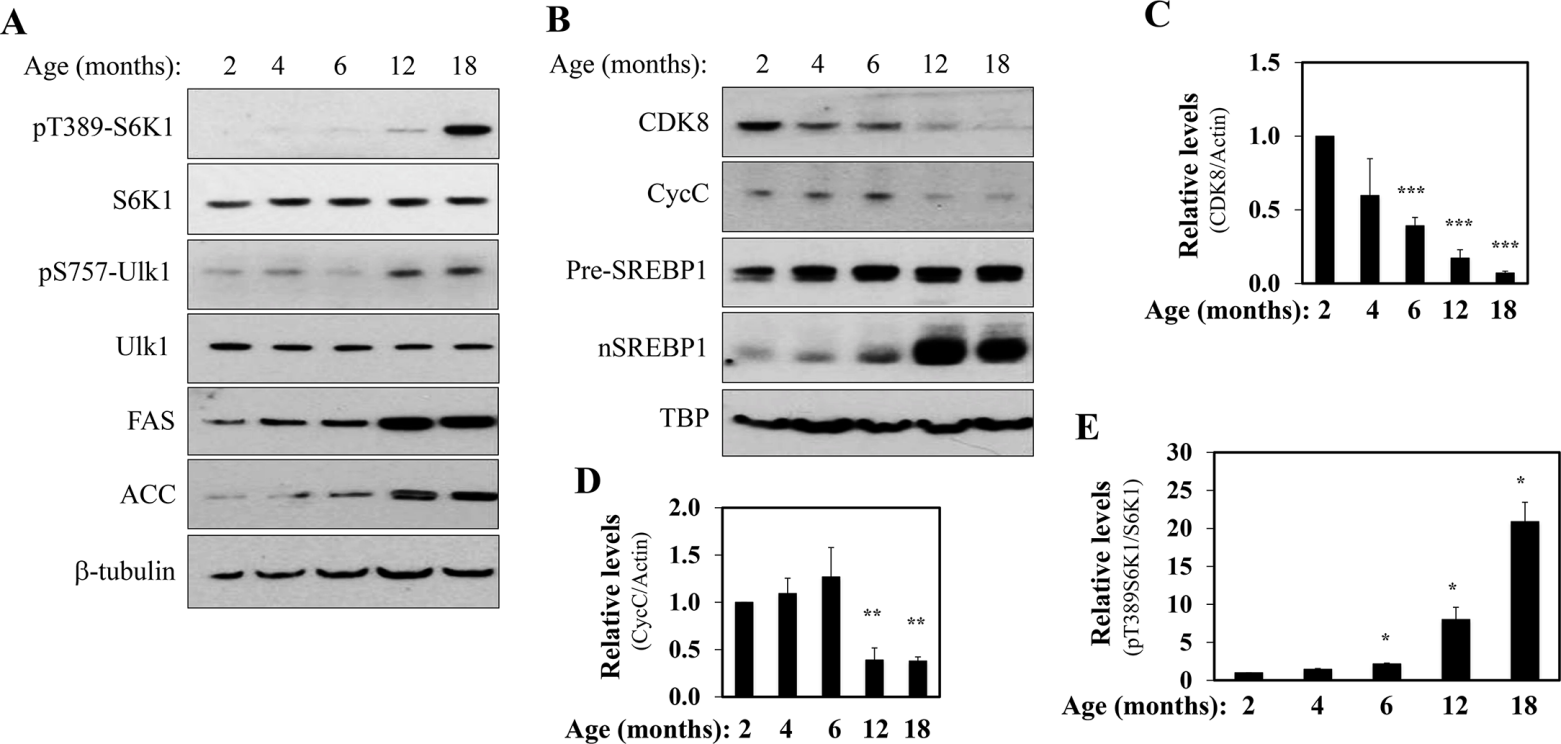
Hepatic levels of CDK8 and CycC proteins are down regulated during aging. C57BL/6J mice were maintained on a normal chow diet and at the ages indicated were fasted for 16 h and liver extracts prepared. **A-B**) Representative immunoblots showing the levels of indicated proteins in livers of mice with indicated ages, as detected by immunoblotting. β-tubulin or TBP served as the invariant controls. Each lane is from the equal pooling of liver extracts from six mice. The relative protein levels of CDK8 (**C**), CycC (**D**) and phosphorylated S6K1 (**E**) were further analyzed by densitometry. *p<0.05, **p<0.01 and ***p<0.001 vs. control (n = 6).

Previous studies have observed that insulin resistance with increased *de novo* lipogenesis occurs during the normal aging process [29–31]. To explore the relationship between mTORC1 activation, the CDK8-CycC complex, SREBP-1c and lipogenic gene expression levels, we examined the protein levels in the pooled mouse livers at different ages (6 mice for each age) by immunoblotting. As shown in Fig 6A, mTORC1 was increasingly activated during aging as detected by the phosphorylation of S6K1 and Ulk1 at the mTORC1-specific sites. This occurred in a coordinate manner with the increase in lipogenic genes, such as FAS and ACC (Fig 6A). In parallel, there was a marked increase in nuclear SREBP-1c proteins with age, while the precursor SREBP-1c protein levels were essentially unaffected (Fig 6B). Strikingly, the protein levels of CDK8 and CycC were decreased in an age-dependent manner (Fig 6B). Image analyses of the semi-quantification of the changes in hepatic protein levels during aging of mice are shown in Fig 6C and 6D. These data demonstrate an inverse relationship between CDK8 and nSREBP-1c in the liver during the normal aging process. Moreover, the age-dependent inverse relationship between hepatic CDK8 and mTORC1 activity was recapitulated in a separate set of pooled livers samples (data not shown).

**Fig 7:**
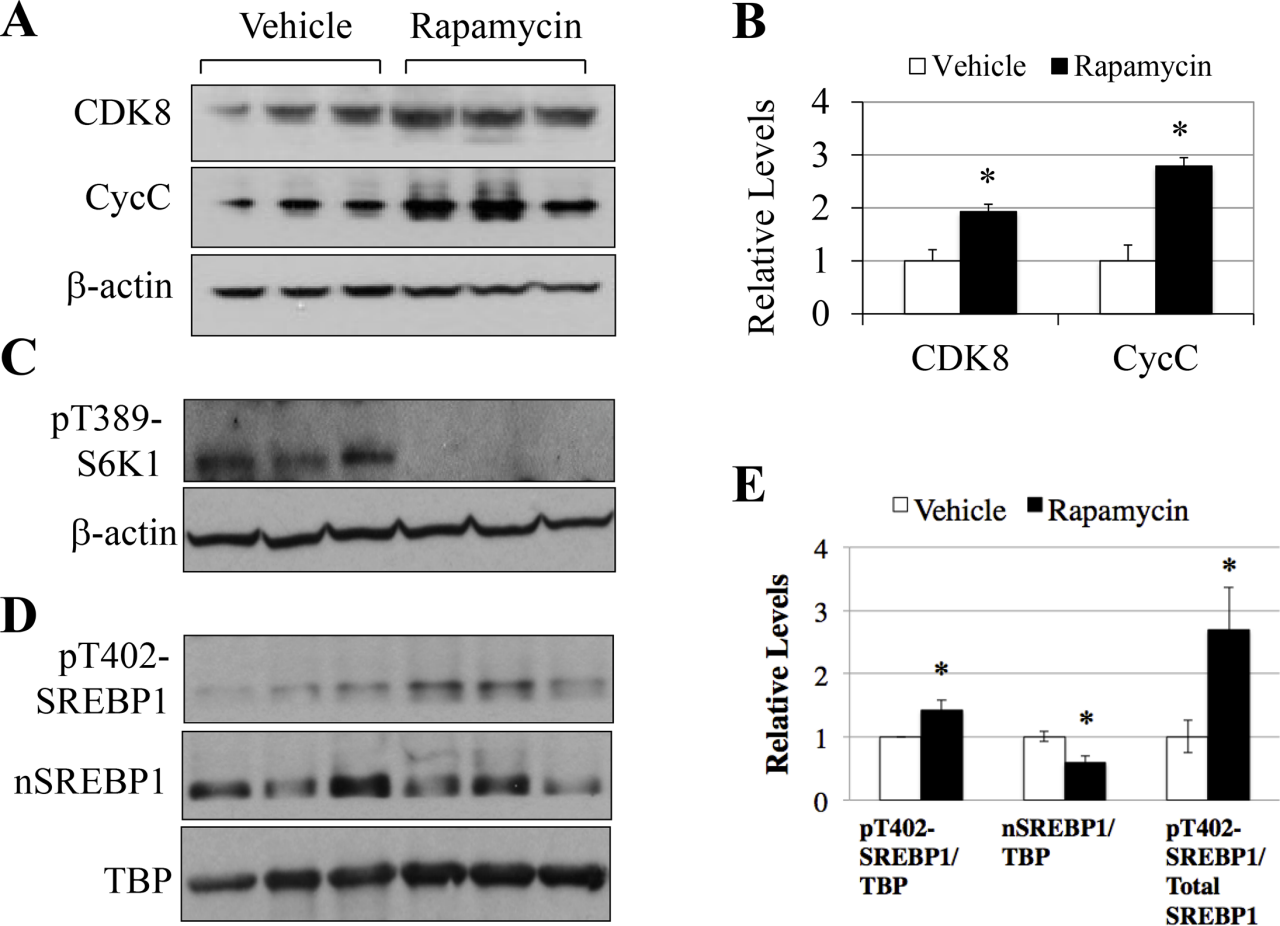
mTORC1 activation is required for the down-regulation of CDK8 and CycC in aging livers. Effects of rapamycin treatment in 12-month old mice on the levels of indicated proteins in the livers, as detected by immunoblotting. β-actin was the loading control. Three independent mice were treated by intra-peritoneal injection of rapamycin (2 mg/kg body weight) or vehicle control for three days. Liver extracts were immunoblotted for (**A**) CDK8, CycC and β- actin, (**C**) pT389-S6K1 and β-actin, (**D**) pT402-SREBP1, nSREBP1 and TBP. **B**) The ratios of CDK8 or CycC to β-actin of indicated treatments were analyzed by densitometry. **E**) The ratios of pT402-SREBP-1/TBP, nSREBP-1/TBP and pT402-SREBP-1/nSREBP-1 of indicated treatments were analyzed by densitometry. *p<0.05 vs. vehicle (n = 3).

To determine whether the age-dependent changes of the CDK8-CycC complex were also due to activation of the mTORC1 signaling, 12 month-old mice were intra-peritoneally injected with the vehicle control or rapamycin for 3 days to inhibit mTORC1. As shown in Fig 7A, rapamycin treatment significantly rescued the protein levels of both CDK8 and CycC in the liver. Semi-quantitative analyses revealed about 2-fold increase of both CDK8 and CycC proteins after rapamycin treatment (Fig 7B). As expected rapamycin treatment inhibited T389 phosphorylation of S6K1, suggesting mTORC1 activation is required for the down-regulation of the CDK8-CycC complex during aging (Fig 7C). Moreover, rapamycin reduced the protein levels of nuclear SREBP-1 (nSREBP-1) while increased the phosphorylation of nSREBP-1 on T402 (Fig 7D and 7E). Thus, these data also demonstrate an important role of mTORC1 in regulating the abundance of the CDK8-CycC protein complex as well as the level of nSREBP-1 in the liver during aging.

## Discussion

In this study, we have shown with multiple lines of evidence that the mTORC1 signaling down-regulates the CDK8-CycC complex at the protein level *in vivo* and *in vitro*. In mouse models of obesity and aging, hepatic mTORC1 is activated and the CDK8-CycC complex is decreased, correlated with the accumulation of nuclear SREBP-1c proteins and lipogenic enzymes. Moreover, the inverse relationship between CDK8 and nuclear SREBP-1c proteins also occurred in human NAFLD. Together, we have identified the CDK8-CycC complex as a novel downstream effector of the mTORC1 signaling and our results suggest an important role of the mTORC1/CDK8 pathway in the development of NAFLD.

Currently, the best-known function of CDK8 is as a reversible subunit of the Mediator complex, which acts as a key cofactor for transcription factors, including SREBP-1c [16, 32] and is conserved among various species [33]. Previous studies have revealed that CDK8 regulates gene expression in both negative and positive manners. Earlier reports showed that the CDK8-containing Mediator inhibits the transcription re-initiation [34, 35]. However, recent studies have shown that CDK8 is also required for context-dependent gene expression [36–40]. While almost all previous studies were to define the functions of CDK8, we have recently shown that CDK8 and its activating partner CycC are down regulated at the protein level by feeding in mouse liver *in vivo* and by insulin in primary rat hepatocytes [10], suggesting that the regulation of the CDK8-CycC complex coordinates the extracellular signals to metabolic gene expression in the nucleus. However, the upstream regulator(s) of the CDK8-CycC complex were not clear. In this study, we have identified mTORC1 as a negative regulator of the CDK8-CycC complex, consistent with the role of mTORC1 as the nutrient/energy sensor and a downstream component of the insulin signaling [20]. Several lines of distinct evidence support this conclusion. First, hepatic mTORC1 activity is inversely correlated with the abundance of the CDK8-CycC complex in three different mouse models of NAFLD. Second, pharmacologic and genetic manipulation of mTORC1 in cell culture alters the levels of the CDK8-CycC complex. Third, liver-specific knockout of Raptor in mice up-regulates CDK8 at the protein levels and blocks feeding-induced down-regulation of CDK8. Finally, acute treatment of aged mice with rapamycin rescues the CDK8-CycC complex. Thus, these data demonstrate that hepatic CDK8-CycC complex is modulated by mTORC1 under physiological conditions.

As a key sensor of cellular energy/nutrient abundance and stress, the mTOR signaling pathway is critically involved in the onset and progression of diabetes, cancer and aging [41]. Recent studies have highlighted the role of mTORC1 in stimulating SREBP1c-dependent lipogenic gene expression and *de novo* lipogenesis [42–48]. mTORC1 is required for insulin-induced up-regulation of *SREBP-1c* transcripts [43], proteolytic cleavage of the SREBP-1c precursor [48], and the control of nuclear SREBP-1c abundance [47]. Since the *de novo* lipogenesis rate is higher in human NAFLD than that of normal subjects [49], our results suggest down-regulation of the CDK8-CycC complex by mTORC1 as an important contributing factor to the observed increase of hepatic *de novo* lipogenesis in NAFLD and insulin resistant states. Although the molecular details of how mTORC1 regulates the CDK8-CycC complex remain unclear, it is likely that phosphorylation is directly or indirectly involved, as mTOR is a serine/threonine kinase. In addition, since mTOR regulates many biological pathways, it will be interesting to identify the biological processes other than *de novo* lipogenesis that are also co-regulated by mTORC1 and CDK8 in future studies.
